# Conservation Actions Based on Red Lists Do Not Capture the Functional and Phylogenetic Diversity of Birds in Brazil

**DOI:** 10.1371/journal.pone.0073431

**Published:** 2013-09-09

**Authors:** José Hidasi-Neto, Rafael Dias Loyola, Marcus Vinicius Cianciaruso

**Affiliations:** 1 Programa de Pós-Graduação em Ecologia e Evolução, Universidade Federal de Goiás, Goiânia, Goiás, Brazil; 2 Departamento de Ecologia, Universidade Federal de Goiás, Goiânia, Goiás, Brazil; University of Toronto, Canada

## Abstract

Red Lists of threatened species play a critical role in conservation science and practice. However, policy-making based on Red Lists ignores ecological and evolutionary consequences of losing biodiversity because these lists focus on species alone. To decide if relying on Red Lists alone can help to conserve communities’ functional (FD) and phylogenetic (PD) diversity, it is useful to evaluate whether Red List categories represent species with diverse ecological traits and evolutionary histories. Additionally, local scale analyses using regional Red Lists should represent more realistic pools of co-occurring species and thereby better capture eventual losses of FD and PD. Here, we used 21 life-history traits and a phylogeny for all Brazilian birds to determine whether species assigned under the IUCN global Red List, the Brazilian national, and regional Red Lists capture more FD and PD than expected by chance. We also built local Red Lists and analysed if they capture more FD and PD at the local scale. Further, we investigated whether individual threat categories have species with greater FD and PD than expected by chance. At any given scale, threat categories did not capture greater FD or PD than expected by chance. Indeed, mostly categories captured equal or less FD or PD than expected by chance. These findings would not have great consequences if Red Lists were not often considered as a major decision support tool for policy-making. Our results challenge the practice of investing conservation resources based only on species Red Lists because, from an ecological and evolutionary point of view, this would be the same as protecting similar or random sets of species. Thus, new prioritization methods, such as the EDGE of Existence initiative, should be developed and applied to conserve species’ ecological traits and evolutionary histories at different spatial scales.

## Introduction

Earth could experience an extreme loss of diversity within a few centuries if current threats to species are not reduced [1]. To overcome this issue, several conservation approaches based on species prioritization are being applied worldwide. The Red List of Threatened Species published by the International Union for the Conservation of Nature (IUCN) is recognized as “the most comprehensive, objective global approach for evaluating the conservation status of plant and animal species” [2]. Red Lists became popular after 1994, when the IUCN defined several scientific criteria to evaluate “if” and “how much” species are endangered worldwide, publishing results periodically. These criteria are based on demographic variables such as rarity, population fluctuations, a species’ extent of occurrence, and a species’ area of occupancy [3]. Following these criteria, when a species is evaluated it can be placed into eight different categories: Data Deficient (DD), Least Concern (LC), Near Threatened (NT), Vulnerable (VU), Endangered (EN), Critically Endangered (CR), Extinct in the Wild (EW), and Extinct (EX) [3].

Red Lists drive national and regional conservation actions and policy in many nations across the globe [4]–[6]. Yet, their validity as conservation and policy instruments has also been a matter of dispute [7]–[10]. One must notice, for example, that Red Lists were designed to indicate the overall risk of species extinction based on demographic variables alone [6], that is, on a species’ extinction probability, not on the consequences of losing a given group of species. Therefore, they do not explicitly incorporate other aspects of biodiversity, such as a species’ genetics, evolutionary history and ecological traits, which is now a clear tendency in conservation literature [11]–[16]. These aspects are important for the maintenance of ecosystem functioning and thus are relevant from a conservation point of view [17], [18].

Traditional measures of biodiversity (e.g., species richness and diversity indices that include evenness) are commonly used together with Red Lists to understand how species and assemblages are being threatened by human activities [19], [20]. However, so far we have no evidence on whether Red Lists are able to protect species’ ecological traits and evolutionary history, and how this inability affects conservation planning [11], [16], [17], [21]–[25]. Moreover, identifying the consequences of species extinction to species’ trait diversity and evolutionary history can help us understand if and how conservation actions can protect multiple components of biodiversity, considering finite resources directed to conservation [26], [27].

Ignoring that species may differ both ecologically and evolutionarily can hide negative human effects on biodiversity. For example, Ernst et al. [28] found that the diversity of ecological traits in anuran assemblages (i.e., functional diversity) was greater in primary than in exploited forests despite the lack of differences in species richness between these forests. Therefore, it is possible to use indices of ‘functional’ and ‘phylogenetic’ diversity, which quantify, respectively, the ecological and phylogenetic dissimilarity among species [29]–[31], in conservation assessments. Functional and phylogenetic diversity could be used to evaluate how natural and anthropogenic disturbances alter assemblages in relation to ecological and evolutionary dissimilarities in their species [32]–[35]. Further, one way to understand the effect of extinctions on biodiversity and assemblage functioning is through simulated extinction scenarios in which assemblages are disassembled using some criteria [32], [36]. Thus, it is possible to use extinction scenarios to simulate the loss of functional and phylogenetic diversity caused by the extinction of threatened species assigned to Red Lists categories. However, the consequence of losing threatened species in terms of their contribution to functional and phylogenetic diversity is still an unanswered question.

Knowing if sets of species assigned to categories of greater concern (e.g., Critically Endangered) on national to regional Red Lists aggregate more functional and phylogenetic diversity than expected from any other set of species can help us understand how and whether Red List categories are able to optimize conservation of the functional and phylogenetic diversity of assemblages [37], [38]. Moreover, regional Red Lists have slightly different criteria for evaluating how close species are to extinction [4]–[6]. Because species are more likely to interact at local scales [39], [40], broad scale analysis can produce null models that include species that do not necessarily co-occur. This model creates a scenario of ‘false complementarity’ in which species artificially compensate for the loss of functional or phylogenetic diversity generated by the loss of species that do not co-occur with them at the regional or local scales. Therefore, the likelihood of Red List categories capturing true losses in functional and phylogenetic diversity would be higher at narrower scales. Hence, we could expect that Red Lists can do better at representing functional and phylogenetic diversity when considering assemblages at regional or local scales.

Brazil holds about 18% of all birds in the world [41]. From these species, about 160 are threatened according to the Brazilian Red List of Threatened Species [42]. The IUCN Red List indicates that over 150 Brazilian bird species are globally threatened [2]. There are also Red Lists for particular individual states in Brazil, which indicate several species that are also threatened regionally [43]–[48]. Here, we used global, national, and regional Red Lists to test if species assigned under different categories concentrate greater bird functional and phylogenetic diversity than expected by chance. We then tested if more threatened categories (e.g., Critically Endangered) capture essential aspects of diversity better than non-threatened categories (e.g., Least Concern). We tested this assumption for the whole country, states, and local sampling sites to evaluate if Red Lists are more likely to capture functional and phylogenetic diversity at narrower scales.

## Materials and Methods

We assessed IUCN categories for Brazilian bird assemblages from national, regional and local scales and considered four categories in this study: Near Threatened (NT), Vulnerable (VU), Endangered (EN) and Critically Endangered (CR). We did not include Data Deficient (DD) because there are no DD bird species in Brazil. Least Concern (LC) species were not considered because they represent the largest number of birds in all lists of species, which would bias our null model results. Also, from our analysed Red Lists, only the global list had LC species. Extinct species (EW and EX) were also excluded from our analysis, because they do not prompt conservation actions.

For national scale analysis, we built a matrix with all non-vagrant birds of Brazil [49] and distributed these species into categories according to the IUCN Red List [50] and the Brazilian Red List of Threatened Fauna [42]. Because the Brazilian Red List does not include NT species, we did not consider this category with this Red List. For regional scale analysis, we overlaid extent of occurrence maps (available at http://www.iucnredlist.org/technical-documents/spatial-data) onto a grid covering a map of Brazil and clipped them together to generate lists of species for six Brazilian states (hereafter ‘regions’) that have their own Red Lists: Espírito Santo [47], Minas Gerais [46], Paraná [45], Rio de Janeiro [43], Rio Grande do Sul [44], and São Paulo [48]. Finally, for local scale analysis, we used local lists of bird species for each of the selected regions. We initially compiled 73 lists, but then only selected lists that had at least two species in at least one IUCN category, the Brazilian Red List or in the respective State Red List. These lists comprise bird assemblages of the Atlantic Forest and Cerrado regions (see Table S2 and Table S3 for more information). We ended up with 48 local lists: four from Espírito Santo, 12 from Minas Gerais, seven from Paraná, five from Rio de Janeiro, six from Rio Grande do Sul, and 14 from São Paulo.

We collated information on 21 ecological traits [16], [32], [51], [52] for 1763 birds of Brazil [49]. We chose ecological traits that distinguish how much species use and compete for resources [38]. In particular, we selected dietary traits (vertebrates, invertebrates, leaves, fruits, grains and nectar; presence/absence), behavioural traits based on foraging methods (pursuit, gleaning, pouncing, grazing, pecking, scavenging and probing; presence/absence) and foraging substrate (water, mud, ground, vegetation and air; presence/absence), period of activity (diurnal and nocturnal; presence/absence), and body mass (in grams; continuous). These ecological traits were also used in previous studies about bird functional diversity [16], [34].

We produced functional dendrograms, which indicate how similar species are in relation to their ecological traits, for the birds of Brazil (national scale), for each of the six regions for which Red Lists were available (regional scale), and for each of the 48 local lists (local scale). To build the dendrograms, we used a modification of Gower’s distance [53] to create a distance matrix from qualitative and quantitative traits, and the unweighted pair-group method using the arithmetic averages (UPGMA) clustering method.

We also made a consensus tree for the birds of Brazil based on 100 phylogenies from birdtree.org [54]. These phylogenetic trees were generated by combining a backbone phylogeny [55] with trees made for species with and without genetic data with the help of taxonomic information and a pure-birth model of diversification, and then assembled based on dated backbone trees and topologies from the literature [54]. We transformed the consensus tree into a cladogram, considering the length of the shorter branches of the cladogram as 1, while other branches were scaled proportionally according to the branching pattern among species. We built the consensus tree using the R software [56] (‘consensus’ function from the ‘*ape’* package; [57]).

For each species pool (Brazilian avifauna, each region’s avifauna and birds from local lists) we measured the amount of functional diversity (FD) [31] and phylogenetic diversity (PD) [29] captured by species within each category (NT, VU, EN, and CR). FD and PD are calculated by summing, respectively, the branch lengths of a functional dendrogram or phylogenetic tree that connect all the species in a given assemblage [29], [31]. For all birds, we calculated FD and PD using the categories from IUCN and the Brazilian Red List, whereas for the regional and local lists we used the IUCN, Brazilian and respective State Red Lists. By doing this, we observed if categories established at different scales (national, regional and local) were able to capture bird FD and PD to a greater extent than expected by chance (see below).

To test whether categories captured more FD and PD than expected by chance, we used null models to compare observed FD and PD values with randomly generated values. At the national scale, we calculated FD and PD values for each category of the IUCN [50] and the Brazilian Red List of Threatened Fauna [42]. At the regional and local scales, we calculated FD and PD based on the IUCN, the Brazilian Red List and the Red List of the respective region. Then, for each category in all analysed scales, we calculated 999 FD and PD values from randomly generated assemblages by shuffling the taxon labels across the tips of the functional dendrogram or cladogram of all species from Brazil or the regional or local lists, holding species richness of each analysed category. Next, we calculated ‘p values’ as the rank of the observed FD or PD value divided by the number of randomized values +1. In that way, for each Red List category, observed FD or PD could be less than (p equal to or less than 0.025), equal to (p between 0.025 and 0.975) or greater than (p equal to or greater than 0.975) than expected by chance.

To test if IUCN, the Brazilian Red List and regional Red List categories from regional and local lists protected, in general, birds that were more functionally and phylogenetically different than expected by chance, we ran Mann-Whitney tests [58] to compare observed FD and PD against the respective mean of randomized FD and PD values. We chose the Mann-Whitney test because the observed and mean values of FD and PD were not normally distributed. If the observed values were less than the mean of randomized values, it indicated that less FD or PD is being captured by species assigned into that category. However, if the observed values were higher than the mean of randomized values, the category captured more FD or PD than expected by chance. Both the null models and the Mann-Whitney tests were done in R software [56] (*‘ses.pd’* function from the Picante package; [59]).

## Results

We observed that IUCN, Brazilian and regional Red List categories assembled different pools of species (see Table S1 for details). Indeed, only the VU and EN categories on the IUCN and Brazilian Red Lists shared more than half of their species. Therefore, categories in the regional lists shared only small numbers of species with both the IUCN and Brazilian Red List categories. At all scales, species assigned to any of these categories did not capture more functional or phylogenetic diversity than expected by chance (see Table 1 and Table 2).

**Table 1:** Percentage of analyzed Brazilian States and sites with threatened birds in categories of the IUCN, Brazilian and respective States Red Lists which had values of observed functional diversity lower, equal or higher than expected by chance (see Supplementary Material for more information).

		Brazilian States				Sites			
Red List	Category	% Lower	% Equal	% Higher	N	% Lower	% Equal	% Higher	N
**IUCN**	**NT**	83.3	16.7	0	6	12.2	87.8	0	41
	**VU**	16.7	83.3	0	6	4.3	95.7	0	23
	**EN**	0	100	0	6	0	100	0	8
	**CR**	16.7	83.3	0	6	NA	NA	NA	0
**Brazilian**	**VU**	0	100	0	6	0	100	0	22
	**EN**	0	100	0	6	25	75	0	4
	**CR**	16.7	83.3	0	6	0	100	0	1
**Regional**	**NT**	0	100	0	2	0	100	0	20
	**VU**	0	100	0	6	2.3	95.4	2.3	44
	**EN**	0	100	0	6	0	100	0	29
	**CR**	0	100	0	6	0	100	0	13

“N” represents the number of States or sites that could be analyzed (had at least one species in the Red List category).

**Table 2:** Percentage of analyzed Brazilian States and sites with threatened birds in categories of the IUCN, Brazilian and respective States Red Lists which had values of observed phylogenetic diversity lower, equal or higher than expected by chance (see Supplementary Material for more information).

		Brazilian States				Sites			
Red List	Category	% Lower	% Equal	% Higher	N	% Lower	% Equal	% Higher	N
**IUCN**	**NT**	83.3	16.7	0	6	9.7	90.3	0	41
	**VU**	33.3	66.7	0	6	21.7	78.3	0	23
	**EN**	33.3	66.7	0	6	0	100	0	8
	**CR**	16.7	83.3	0	6	NA	NA	NA	0
**Brazilian**	**VU**	16.7	83.3	0	6	22.7	72.7	4.5	22
	**EN**	0	100	0	6	25	75	0	4
	**CR**	0	100	0	6	0	100	0	1
**Regional**	**NT**	0	100	0	2	0	95	5	20
	**VU**	50	50	0	6	20.4	77.3	2.3	44
	**EN**	50	50	0	6	20.7	79.3	0	29
	**CR**	33.3	77.7	0	6	7.7	92.3	0	13

“N” represents the number of States or sites that could be analyzed (had at least one species in the Red List category).

At the national scale, two categories (NT and VU) from the IUCN Red List assembled birds that were functionally more similar than one would expect by chance, while all categories in the Brazilian Red List had a random loss of FD (see Table S2 for details). Also, all categories on the IUCN Red List and one category from the Brazilian Red List gathered birds that were phylogenetically more similar than expected by chance (see Table S3 for details). Moreover, when considering IUCN, National and regional Red Lists, we found that most analysed regions and sites had threatened birds with FD equal to that expected by chance.

Indeed, at the regional scale, only the NT category of the IUCN Red List assembled birds that represented losses in FD and PD less than those expected by chance in most regions (83.3%). For regional Red Lists, when considering NT species, 10.4% of sites had less FD than expected by chance. When considering the VU category, 66.7% of regions and 18.75% of sites had less PD than expected by chance. Nevertheless, at the local scale, we found greater FD than expected by chance for VU species in only one site (located in São Paulo), and greater PD than expected by chance for NT species in another site in São Paulo. Likewise, we found that one site located in Rio Grande do Sul had VU species, listed in both in the Brazilian and Rio Grande do Sul Red Lists, with greater PD than expected by chance.

Not surprisingly, we found that, on average, IUCN categories gathered species that had FD and PD levels similar to what one might expect by chance (Table 3 and Table 4). Indeed, most of the Mann-Whitney results showed no difference between the observed FD and PD and the mean of randomized values for the categories on both the regional and local scales. However, we found that for the analysed regions, birds in the NT category of IUCN Red List were, on average, more similar in relation to their ecological traits than expected by chance (i.e., functional clustering). Likewise, among local lists, birds in the EN category of the regional Red Lists were, on average, more similar in relation to their evolutionary history than expected by chance (i.e. phylogenetic clustering).

**Table 3:** Results for the Mann-Whitney tests comparing values of observed functional diversity and mean of randomized functional diversity of the analyzed Brazilian States and sites with threatened birds in categories of the IUCN, Brazilian and respective States Red Lists (see Supplementary Material for more information).

			Brazilian States			Sites		
Red List		Category	U	P	N	U	P	N
**IUCN**		**NT**	4,000	0.025	6	740.000	0.351	41
		**VU**	13.000	0.423	6	244.000	0.652	23
		**EN**	13.000	0.423	6	14.000	0.059	8
		**CR**	10.000	0.200	6	NA	NA	0
**Brazilian**		**VU**	13.000	0.423	6	237.000	0.907	22
		**EN**	15.000	0.631	6	4.000	0.248	4
		**CR**	13.000	0.423	6	NA	NA	1
**Regional**		**NT**	NA	NA	2	197.000	0.935	20
		**VU**	14.000	0.522	6	962.000	0.960	44
		**EN**	15.000	0.631	6	401.000	0.762	29
		**CR**	16.000	0.749	6	70.000	0.457	13

“N” represents the number of States or sites that could be analyzed (had more than two species in the Red List category). Statistically significant values are underlined.

**Table 4:** Results for the Mann-Whitney tests comparing values of observed phylogenetic diversity and mean of randomized phylogenetic diversity of the analyzed Brazilian States and sites with threatened birds in categories of the IUCN, Brazilian and respective States Red Lists (see Supplementary Material for more information).

			Brazilian States			Sites		
Red List		Category	U	P	N	U	P	N
**IUCN**		**NT**	7,000	0.078	6	734.000	0.323	41
		**VU**	15.000	0.631	6	206.000	0.199	23
		**EN**	16.000	0.749	6	26.000	0.529	8
		**CR**	10.000	0.200	6	NA	NA	0
**Brazilian**		**VU**	17.000	0.873	6	216.000	0.542	22
		**EN**	12.000	0.337	6	6.000	0.564	4
		**CR**	13.000	0.423	6	NA	NA	1
**Regional**		**NT**	NA	NA	2	191.000	0.808	20
		**VU**	12.000	0.337	6	817.000	0.208	44
		**EN**	15.000	0.631	6	293.000	0.047	29
		**CR**	15.000	0.631	6	66.000	0.343	13

“N” represents the number of States or sites that could be analyzed (had more than two species in the Red List category). Statistically significant values are underlined.

## Discussion

There is an increasing body of evidence that FD is positively related to ecosystem functioning and stability [14], [30], [60]. Also, PD is often a proxy for FD [61]–[63] and for species evolutionary potential, known as the potential capabilities of species to evolve given environmental changes [64]. Thus, the conservation of such components of biodiversity might be important for maintaining ecosystem processes and services, and must be set as a goal in conservation planning [14], [25], [37], [64], [65].

We found, however, that Red List categories did not capture greater FD or PD than expected by chance. Because the objective of Red Lists is species and population recovery as opposed to the conservation of other aspects of biodiversity (e.g. functional or phylogenetic diversity), it is no surprise that species within given IUCN categories do not capture more FD or PD than expected by chance. This is a crucial point, because Red Lists (and therefore rarity) are often the only information considered in national conservation assessments and planning [10], [66]. Still, we only found greater FD for VU species in a single site in São Paulo, and greater PD for NT species in another site in São Paulo, and for VU species in a site in Rio Grande do Sul (Table S2 and Table S3). Despite not being much threatened today, these species have important ecological and evolutionary roles. This is the typical case where species with important ecological roles that might suffer rapid population declines [67], [68] are not appropriately targeted for conservation purposes. For example, it has been demonstrated that common species with important ecological roles in their native ranges, like the common starling (*Sturnus vulgaris*) in Europe, suffered from dramatic population losses in recent decades [67]. If most of the national budget for biodiversity conservation is allocated to the protection of threatened species, we may face the risk that, in the long term, we will lose ecosystem functions and services because we neglected the functional role of species when defining our national conservation action and policy.

Extinctions are not expected to occur at random [36], [69]. Indeed, Szabo et al. [70] found that extinctions were disproportionally concentrated in species-poor bird families. Then, some ecological traits can be more closely related than others to the extinction of species, revealing patterns of functional vulnerability among Red List categories. We found that the NT category of the IUCN Red List represented fewer losses of FD and PD than expected by chance in most of the regions. In other words, this category concentrates species that share most of their ecological traits and evolutionary history. These ecologically ‘redundant’ species are sometimes referred to as maintainers of reliable ecosystem functioning, and thereby are important from a conservation perspective [71]. Species with similar evolutionary histories may share a large number of ecological traits [72], so a portion of these results can be explained by a phylogenetic signal in species traits already captured by our previous finding for FD (see above). When considering the VU category on regional Red Lists, we also found that 66.7% of regions had less PD than expected by chance. In this way, at the regional scale, the NT and VU categories concentrated, in general, more recent species that share a large amount of their evolutionary history and might have high evolutionary potential, which is the ability of species to evolve in the face of environmental changes [62]. This finding is contrary to Purvis et al. [36], where the simulated extinction of endangered species represented a greater loss of PD than expected by chance for mammals, birds and primates at the global scale. We observed Red List categories with threatened species that have similar traits and share a large amount of their evolutionary history, which are the likely intrinsic biological drivers of their extinction. Hence, that reinforces our need to identify which traits are more related to a species’ probability of extinction [24] and which clades are more likely to disappear in the future [36].

Species are more likely to interact with each other on local scales [39], [40]. Therefore, broad-scale analysis can produce models in which species are ‘falsely complementary’ to each other in relation to their functional traits or evolutionary history. For example, when considering all the birds in Brazil for analysing the functional or phylogenetic diversity of threatened species, birds from different ecoregions or biomes are treated as if they would always occur together. If these species are ecologically or phylogenetically similar and some of them become extinct, then the remaining species will compensate for the functional/phylogenetic losses. Thus, narrow scales would better represent the consequences of losing endangered species. Indeed, we only found greater FD and PD than expected by chance in a few categories of less concern at the local scale (as we discussed previously). However, even after reducing the effect of ‘false complementarity’ among species in relation to their ecological traits and evolutionary history at narrow scales, Red Lists, in general, were still not able to protect more functional and phylogenetic diversity in any of the analysed scales.

Brito et al. [6] found for Brazil, China, Colombia and Philippines a considerable number of species that were listed as threatened by the IUCN, but were not listed nationally (average of 14%) or that were listed as threatened nationally but not assessed by the IUCN (average of 20%). Moreover, it is known that the overall IUCN threat status of the birds of the world has deteriorated since 1988 [73], meaning that species from categories of less concern have been moved to ones of greater concern. We observed that different assessments, independent of their categories, were equally incapable of protecting more FD or PD than expected by chance. We observed that categories in the IUCN, national and regional Red Lists did not contain the same species (see Table S1). In fact, the similarity within categories among these lists ranged from 0 to 76%. This reinforces our finding that these categories did not capture bird FD and PD, and also highlights that our congruent results for FD and PD were not due to a possible high species similarity among categories from different Red Lists.

Measures of ecological and evolutionary dissimilarity can be implemented into extinction risk assessments [21], [64], [65], [74]. They represent the relative contribution of threatened species to the PD and FD of assemblages. For example, there is an initiative for protecting species that are evolutionarily distinct and globally endangered according to the IUCN Red List, known as EDGE [11]. Also, it is known that indicator groups that represent the dissimilarities in both ecological traits and evolutionary history should be conserved, maintaining ecosystem processes and evolutionarily distinct species [16]. These studies can bring us cost-effective shortcuts for conserving assemblages’ ecological and evolutionary dissimilarity while protecting globally or regionally threatened species. However, as noted by Winter et al. [74], we still need a guideline for including species PD in conservation, and other components of biodiversity, such as FD, should be included whenever possible. In that way, there is some debate on whether and how we should target less or more FD and PD. We found several cases in which Red List categories protected sets of functionally redundant (less FD than expected by chance) and closely related species from more recent clades (less PD than expected by chance). It is important to note that these species might be able to protect both the maintenance of ecosystem functions and services [71] and the evolutionary history of recent clades due to their high evolutionary potential [62].

As resources directed to conservation are finite, it is important that both proactive and reactive actions consider multiple variables, like costs, benefits (e.g., ecosystem functions and evolutionary history), species extinction probability, and likelihood of management success [26], [27]. The failure of Red Lists to capture PD and FD highlights our need for new methods for prioritizing species’ ecological and phylogenetic uniqueness [11], [18], [27] and inform us about the consequences of species loss to ecosystem processes instead of using only demographic variables to understand which species are more likely to disappear first.

## Supporting Information

Table S1 **Richness and similarity of species composition of similar categories among IUCN, Brazilian and regional red lists.** IUCN categories have the following species richness: NT = 95; VU = 65; EN = 35; CR = 21. Categories that share more than a half of their species have values in bold.
(XLS)

Table S2 **Relation between observed functional diversity values and the mean of randomized functional diversity values for six Brazilian regions and 48 sites; and the reference for the sites and the status of conservation of these areas, as well as the total number and the number of threatened birds within each Red List category for Brazil, each State and each site.**
(XLS)

Table S3 **Relation between observed phylogenetic diversity values and the mean of randomized phylogenetic diversity values for six Brazilian regions and 48 sites; and the reference for the sites and the status of conservation of these areas, as well as the total number and the number of threatened birds within each Red List category for Brazil, each State and each site.**
(XLS)
